# Review of the annual reports on antibiotic policy data from hospitals, private clinics, and rehabilitation facilities in Paris city

**DOI:** 10.1186/2047-2994-4-S1-P184

**Published:** 2015-06-16

**Authors:** JF Westphal

**Affiliations:** 1 Public Health Department, Regional Agency for Health, Paris, France

## Introduction

Each hospital, private clinic (PC), or rehabilitation facility (RF) in France has to produce a computer-based annual report on the organization and actions developed for its antibiotic policy to the French Ministry for Health. This report consists of a list of nationally pre-defined items that the physician responsible for the infection control unit has to fill online. A scoring computer program operates at the end of the process.

## Objectives

The aim of the study was to analyze the results of the annual report provided by every hospital, PC, or RF in Paris city in 2014.

## Methods

Assessment of the reports was performed by the Regional Agency for Health Ile-de-France, a dependency of the French Ministry for Health.

Evaluation data included the general grade (decreasing from A to E) and the review of all the items structuring every annual report.

## Results

The reports of 69 healthcare organizations (comprising 17,483 full-time hospitalization beds) were examined, including 17 university hospitals (UH), 16 public general hospitals (GH), 25 PC, and 11 RF.

Distribution of the classes A and B taken together was 65% in UH, 87% in GH, 92% in PC, and 82% in RF.

Results appeared to be perfectible in the items dealing with the means dedicated to prevention and control activities: only 47% of UH and 36% of PC had a full computerized physician drug-order entry system, and more than one third of PC or RF had no antibiotic therapy specialist.

In contrast with better results from the other healthcare organizations, respectively, 35% of UH and 59% of UH had no defined collaboration between the antibiotic specialist, the pharmacist, and the microbiologist, and had no list of selected antibiotics under increased control.

Less than two thirds of GH or RF complied with the items dealing with antibiotic utilization evaluation.

## Conclusion

Analysis of the annual reports from all the hospitals, clinics, and rehabilitation facilities in Paris city on the nationally-required activities in antibiotic policy highlighted relatively good global results since over 80% of all these organizations taken together was graded A or B. However, the results in those items dealing with the means allocated to prevention of antibiotic misuse or with evaluation of antibiotic prescribing practices need to be improved.

## Disclosure of interest

None declared.

